# Introducing participatory fairness in emergency communication can support self-organization for survival

**DOI:** 10.1038/s41598-021-86635-y

**Published:** 2021-03-30

**Authors:** Indushree Banerjee, Martijn Warnier, Frances M. T. Brazier, Dirk Helbing

**Affiliations:** 1 TU Delft, Systems Engineering and Simulations, Delft, The Netherlands; 2 ETH Zurich, Computational Social Sciences, Zurich, Switzerland

**Keywords:** Complex networks, Natural hazards

## Abstract

Participatory resilience of disaster-struck communities requires reliable communication for self-organized rescue, as conventional communication infrastructure is damaged. Disasters often lead to blackouts preventing citizens from charging their phones, leading to disparity in battery charges and a digital divide in communication opportunities. We propose a value-based emergency communication system based on participatory fairness, ensuring equal communication opportunities for all, regardless of inequality in battery charge. The proposed infrastructure-less emergency communication network automatically and dynamically (i) assigns high-battery phones as hubs, (ii) adapts the topology to changing battery charges, and (iii) self-organizes to remain robust and reliable when links fail or phones leave the network. The novelty of the proposed mobile protocol compared to mesh communication networks is demonstrated by comparative agent-based simulations. An evaluation using the Gini coefficient demonstrates that our network design results in fairer participation of all devices and a longer network lifetime, benefiting the community and its participants.

## Introduction

*“How effective are existing innovative ways to share data in humanitarian settings, such as mesh networks, bluetooth technology, microwave technology and peer-to-peer networks? What other novel strategies exist?”* From: “Grand Challenges of Humanitarian Aid”, Nature, 2018^1^.

We are living in a world in which the frequency and severity of natural disasters—causing deaths and displacements—are steadily increasing^2^. This is also because of tipping points^3^ and cascading effects^4,5^ in complex anthropogenic systems^6^. The aftermath of Hurricane Katrina, the Nepal earthquake, and the Indian Ocean tsunami has shown that delays in rescue operations lead to the loss of additional human lives^1^. The number of casualties could be reduced if interventions such as preliminary first aid and basic support were provided during the first 72 h following a disaster, called the “Golden Period”^7^. However, mobilizing rescue operations and professional help for disaster recovery takes time^7,8^. It is, therefore, crucial that citizens are provided with tools that enable participatory resilience and sustainability, allowing them to help themselves and support each other^7,9,10^.

Emergency citizen-based smartphone applications enable communication and collaboration during the aftermath of a disaster, thereby supporting community resilience. Simultaneously, reliable communication in an uncertain and dynamically changing environment^11^ is challenging^12^. The challenge to stay connected during disasters is increased by (i) failure of telecommunication infrastructures due to damage, and (ii) limited battery charge in phones due to power blackouts^13–18^. Power grids and mobile telecommunication are highly interdependent, so the failure of one has a cascading effect on the other^19,20^.

For example, 8000 mobile base stations immediately failed in Japan on March 11, 2011 after the tsunami. This number doubled by the following day, as backup power was exhausted, which led to 85% of mobile communication breaking down during this time^21^. Hurricane Katrina damaged three million telephone land-lines, disabling numerous 911 call centers. With approximately 2000 cell sites uprooted and limited locations to charge phones due to power outages, many wireless phones were not reachable^13^.

This has led to the development of various smartphone applications that are promoted as facilitating emergency communication^22,23^. In recent years, there has been a rise in designing autonomous, self-organising mobile ad-hoc networks that use leftover technology or smartphones^24–27^. These applications utilize wireless capabilities of end-user devices such as Bluetooth and Wi-Fi to exchange messages peer-to-peer, forming an “ad hoc” communication network on-the-fly^28–30^.

In these applications, there is typically a direct point-to-point connection between all phones that are in transmission range of each other. If sender and receiver are not within the transmission range, the message is relayed by other phones. This connection pattern is termed a mesh topology^29^. A mesh topology is the standard connection pattern for existing generic applications such as TeamPhone^31^, RescueMe^32^, FireChat, ServalMesh^33^, BATMAN^34^, Twimight^28^ and Bluemergency^35^.

The use of ad hoc networks for emergency communication has been around for the past 40 years. Still, its effectiveness is limited as long as data access is not democratized^1^. In particular, unavailability of charging facilities creates a disparity in phone battery charge, limiting communication for a considerable fraction of affected citizens. This digital divide can be fatal during an emergency, when communication is vital for survival.

We make the right to communicate and remain connected despite a disparity in battery charge the core of our design process and show that it is possible to overcome the related digital divide. With our ethically aligned, value-sensitive design we introduce a novel emergency communication system called “Self-Organisation for Survival” (“SOS”). SOS promises affected communities to have extended and increased access to communication via a peer-to-peer communication network that is designed for fair participation. Extending the current body of work on citizen-based ad hoc networks for crisis and disaster, we offer a solution to one of the grand challenges of humanitarian aid^1^.

Our definition of “participatory fairness” is distinct from the fairness principles used in the areas of computer networks and resource scheduling^36–38^. The principle of fairness in computer science has been studied for the past 30 years with a fairness index^39^. This index measures the “equality” of user allocation of resources. Fairness as a concept has also been introduced in wireless networks related with fair channel allocation, bandwidth and throughput allocation^40,41^. The goal has been to deliver fair end-to-end performance in wireless multi-hop networks^42^. These definitions, however, are not relevant for our work, as we are interested in *social* fairness and its individual and collective benefits.

Our system strives towards the creation of a collective public good and its fair use. In an emergency, the system design should not lead to a discriminatory bias against people with less battery charge and related communication resources. We, therefore, define fairness in the sense of equity and social justice.

This paper goes beyond the current state of the art in three ways: First, we introduce a value-sensitive design approach for communication networks. Second, we boost resilience by introducing participatory fairness into the operation of a peer-to-peer network based on context-adaptive self-organisation. Third, we improve the energy efficiency of communication under stress to benefit disaster-struck communities over extended periods of time, when they need communication most to help each other and survive. Overall, this creates massive individual and collective benefits.

In the following, we discuss the implementation and advantages of a value-sensitive design called “Self-Organization for Survival” (“SOS”), which is specifically made for disaster scenarios. It can benefit individuals and promote collective behavior based on local interactions^43^. Compared to a typical mesh network, the design of SOS ensures that phones with different battery charge have the opportunity to communicate for 72 h without recharging. It does so by considering the additional value of “participatory fairness”.

### Design for values

“Design for values” is an approach to include values such as autonomy, fairness, usability, privacy, or democracy in the design and operation of technology^44,45^. Existing applications establishing an infrastructure-less mesh network are implicitly or explicitly designed for citizen-based communication. They provide autonomy from a backbone infrastructure and reliability in communication. To facilitate collaboration and communication of a community of people during an unexpected disaster, however, other factors must be considered as well. These include the unavailability of charging opportunities for participating devices, and the amount of time phones must be able to communicate, while the network needs to continuously adapt to a changing environment.

If an application is not designed with these factors in mind, biases may arise that could compromise the outcome in three ways^46^: through preexisting biases, technical biases, and/or emergent biases. Preexisting biases are rooted in the fabric of society. Technical biases refer to technical constraints or issues. Emergent biases result from the usage, which may depend on the context. Today’s ad hoc networks have technical biases due to technical limitations and emergent biases due to lack of consideration. Technical restrictions such as lack of charging facilities and limited resources of phones may also imply emergent biases such as the disparity of communication opportunities. We address the issue of participation disparity in the following.

Our paper pursues a design-for-values approach to reduce technical and emergent biases in a dynamically changing context. We have, therefore, designed a context-adaptive distributed protocol that uses local self-organisation to achieve *participatory fairness* in a peer-to-peer communication network. SOS enables and maintains the participation of practically *all* phones, i.e., it provides equal communication opportunities for all citizens regardless of the initial inequality in phone battery charges. Overcoming inequality serves to keep the social fabric functional under stress, for example, during crisis and disasters.

### SOS: designing for “participatory fairness”

A phone loses battery charge when connecting to another phone or when sending, receiving, or relaying a message (see SI for realistic values). For simplicity, in our agent-based models and computer simulations, these costs are assumed to be the same for all phones participating in the formation of the peer-to-peer (“ad hoc”) communication network. Note, however, that the battery charge is different, since some phones will have recently been charged, while others are about to run out of power. In addition, different phone models have different battery capacities. This disparity of phone battery charge would usually imply the loss of connectivity and communication opportunities over time for a quickly increasing number of phones. To ensure participatory fairness, we propose a communication protocol that avoids unnecessary connections and relays messages in a way that is, in a sense, proportional to battery charge.

**Figure 1:**
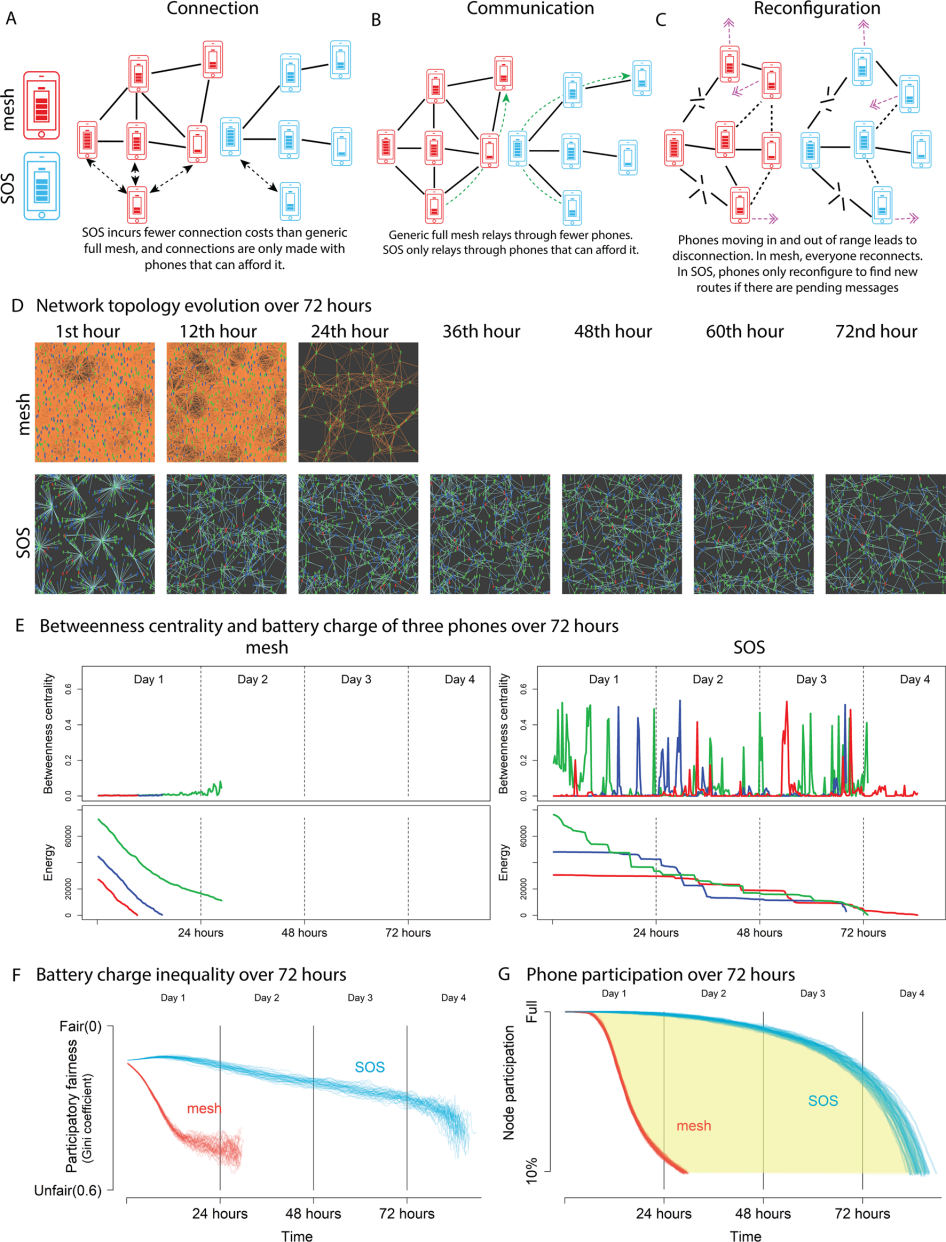
(**A**) Differences in connection, (**B**) communication and (**C**) reconfiguration patterns between a generic mesh protocol (red) and SOS (blue). (**D**)–(**G**) Results of simulations with 500 phones, sending and receiving 1 message per phone every 15 min for the generic mesh and SOS protocols. (**D**) Formation and evolution of the mesh (top) and SOS topology (bottom). The ad hoc mesh network runs just longer than a day. SOS runs for the entire duration of 72 h. (**E**) Development of Battery charge (Energy) and Betweenness Centrality for a selection of three typical phones (red: low initial battery charge; blue: average initial battery charge; green: high initial battery charge) Left: mesh network; right: SOS. In the mesh network, every phone has the same Betweenness Centrality. In SOS, the Betweenness Centrality fluctuates, with green starting as a central hub; a role which is later taken over by blue and then red, as the relative battery charge changes. This ensures that all phones can equally participate in communication for an extended time period. (**F**) Development of battery charge inequality (Gini coefficient^52^) over 72 h for the mesh network (red) and for SOS (blue). (**G**) Phone participation over 72 h for the mesh network (red) and for SOS (blue).

In conventional mesh networks, every phone connects to every other phone in the transmission range (Fig. 1A; red phones). This results in direct peer-to-peer connections and few relays, but connection costs quickly increase with population density. Moreover, even low-battery phones may be used to relay messages (see Fig. 1B, red phones).

Using a minimal spanning tree for communication instead will reduce the number of links and, thereby, connection costs. However, it will increase relay costs to route all messages from the senders to the respective receivers. Both approaches will quickly discharge low battery phones, which will lose their communication opportunities quickly.

We overcome this problem by designing the “SOS” protocol for participatory fairness. For this, high-battery phones capable of maintaining a large number of connections are automatically assigned as hubs, leaving the low-energy phones with fewer connections and lower relay costs. As time progresses and high-battery hubs lose energy, they automatically change position within the peer-to-peer communication network, and nodes that then have higher battery charge become the new hubs (see Fig 1E).

SOS uses (1) the principle of self-organization to maintain participatory fairness and (2) distributed local information exchange to learn about the spatio-temporal context and resources. To be context-aware, every phone gathers local information about other phones in its transmission range (which consumes energy as well, as considered by our model). The local information consists of the battery charge of its neighbors as well as whether neighbors are part of an existing network. Once this information is exchanged, phones follow two rules before getting connected: (i) select the phone with the highest battery charge in range for possible connection (ii) connect only if the phone is part of a different network, i.e., do not connect to a phone, if intermediary connections already exist. If there are messages to send and no route is present, the SOS protocol will reconfigure the local connections (see the Supplementary Information for details of the corresponding Algorithms). Overall, these rules lead to the emergence of a peer-to-peer network with the following characteristics:

- as a result of rule (i), phones with a high battery charge have a higher connectivity, while phones with a low battery charge automatically become leaf nodes with a single connection to the network (see Fig. 1A; blue phones).
- as a result of rule (ii), multiple connections and related costs are avoided.
- the topology is adaptive: It accounts for changes in the local context and updates the roles of participating nodes, if needed.

No manual intervention is required to adapt the topology, since the reconfiguration is event-driven (see Fig. 1C; blue phones). The distributed message exchange and context awareness make the network scalable and adaptable to changes of the density or mobility of people. Complementary, a detailed technical performance analysis of the SOS approach has been performed in a previous paper^47^, evaluating the effects of varying density and message frequency on message reliability (and scalability and longevity). There, it has been found that the reliability remained above 80% under a wide range of conditions.

Further explanations of functional and non-functional requirements, subsequent design choices and their implications as well as the pseudo-code of the SOS protocol are presented in the SI.

## Results

We use an agent-based modeling^48^ approach to compare the SOS approach to a generic mesh network. For the results of Fig. 1, we simulate a torus-shaped world of 25 × 25 units with 500 people having phones, assuming normally distributed battery charges (see SI for more details). In our model, people with phones perform a random walk moving at constant speed (which simplifies typically observed mobility patterns^49^). For simplicity, each phone sends one message every 15 min to a randomly chosen other phone (even though the real message frequency is not this homogeneous^50^). The transmission range is assumed to be homogeneous at 5 units. The loss of battery charge associated with sending, receiving, relaying, connecting and reconfiguring is specified according to real Bluetooth Low Energy battery costs^51^.

**Figure 2:**
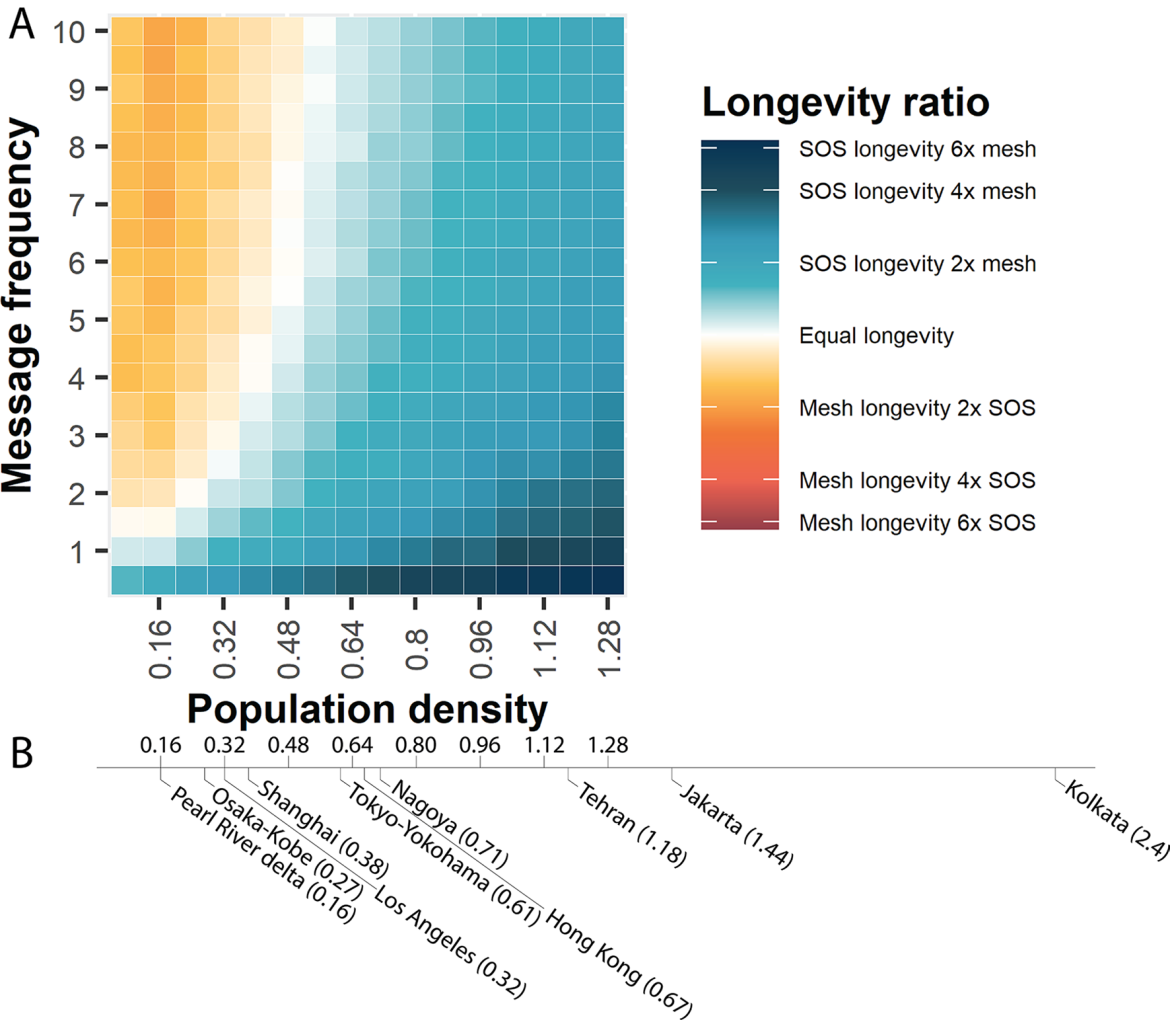
(**A**) Phase diagram of the difference in longevity between mesh and SOS, for varying message frequency and population density. Light blue to deep blue indicates an increasing advantage for SOS over mesh. Orange to red indicates an increasing advantage for mesh over SOS (dark red not occurring). White indicates that longevity was equal between the two. SOS performs best when population densities are higher (towards the right) or message frequencies are lower (towards the bottom). Mesh performs best when population densities are low and message frequencies are high. (**B**) Top ten cities (and their density) in the world that have the highest risk of getting affected by natural calamities.

For the results of Fig. 2A, settings were the same as above, with the exception of the number of people and message frequency. The number of people was systematically varied from 100 to 800 to estimate the effect of population density on longevity. Message frequency was varied between one and ten messages sent every 15 min, to estimate the effect of the amount of data traffic on longevity.

### SOS lasts for 72 h and considerably longer

Figure 1D (and movie S1 in SI) shows the development of the topology over 72 h for the mesh and SOS communication topologies. The mesh topology is tightly coupled with peer-to-peer connections between phones in each others’ transmission range in the first hour. This continues and results in a crowded topology for 24 h. Despite phones moving in and out of range, there is no noticeable change in topology. This is due to the “connect to all in range” characteristics of mesh.

The topology that emerges due to the SOS algorithm is context-adaptive and self-organised, with a few hubs and many low-degree nodes. This is most clearly visible in the 1st hour. Some phones (with a high battery charge) have many phones connected to them, acting as hubs. Others (with a low battery charge) have one connection and lie on the edge of the network. Initial high battery charge phones later on take less central positions in the network, when other high battery charge phones take over as hubs. Over time, this results in a network with a more even energy distribution among phones.

### SOS adapts the traffic distribution to spare low-energy phones

Figure 1E (and movie S2 and S3 in SI) shows how the adaptive mechanism of SOS affects the consumption of battery charge over time and the Betweenness Centrality. Betweenness Centrality measures the importance of a phone for passing information. Higher Betweenness Centrality shows that a particular phone is more centrally placed in the emerging network, which means maximum data traffic passes through this phone.

For mesh networks (see left of Fig. 1E), the battery charge rank of phones is stable over time. All phones have almost the same Betweenness Centrality with a slight variation at the end towards 0.14, and all phones lose battery charge linearly over time. This creates a discriminatory bias against people with phones that happened to have a low initial battery charge (such as the red phone). They are disconnected earlier, limiting their communication opportunities in favour of people with a higher battery charge (green).

SOS automatically assigns high-energy phones as hubs and monitors the spatio-temporal energy distribution to adapt the network topology. This mechanism prevents selfish behaviour and promotes altruism, which is reflected in the changing Betweenness Centrality. For the SOS protocol, the figure on the right of Fig. 1E shows how the role of phones changes over time. The green phone initially plays a central role (with a Betweenness Centrality of 0.52 after 2 h), because it has a high level of battery charge, while other phones are spared. After some time, the blue phone becomes a hub (with a Betweenness Centrality of 0.54, peaks between hours 25 and 38, and again towards the end). After that, there is a period where the red phone becomes a hub (with a Betweenness Centrality of 0.53).

The red phone starts with the lowest battery charge and is the first to disconnect in the mesh network. In SOS, the red phone is spared from relaying messages, allowing it to stay connected for as long as the green phone with the highest initial battery charge. This illustrates how the topology adapts to the spatio-temporal situation of energy availability. The phones keep changing with regard to the load and traffic, to spare the lower battery charge phones, such that participatory fairness is achieved.

### SOS distributes energy more fairly over phones than traditional mesh

The participatory fairness of the phone battery charge distribution is calculated here with the Gini coefficient. Figure 1F shows the Gini coefficient over time, for SOS (in blue) and mesh (in red). The Gini coefficient is typically used to study inequality, e.g. of income or resources^52^. Its value ranges from 0 to 1 with 0 signifying complete equality (all have the same battery charge) and 1 meaning extreme inequality (one phone has all battery charge).

For the traditional mesh network, inequality increases quickly, with the Gini coefficient increasing from 0.13 to 0.39 within the first 14 h, then stabilizing around 0.45. For SOS, inequality decreases within the first 10 h, with the Gini coefficient decreasing from 0.13 to 0.11. Then, within the next 62 h, the Gini coefficient slowly increases to 0.27.

### SOS allows more phones to participate for a longer period than mesh

Figure 1G (and movie S1 in SI) shows phone participation over time. For mesh, phones almost immediately start to fail with the first phone dropping out after 3 h. For SOS, the time period during which there are no failing phones is significantly extended, with the first phone dropping out of the network after 13 h. Also, a significant improvement in longevity for SOS is immediately obvious, reflected by the considerable horizontal distance between the SOS and mesh curves. For the mesh topology, only 18% of phones remain connected after 24 h, whereas for SOS, 99% of phones are still connected after 24 h. For SOS, the phone participation is recorded to be 91% after 48 h and 62% after 72 h.

This illustrates a large difference in the energy efficiency between the two protocols. SOS has several advantages: The communication network lasts considerably longer, and the percentage of phones participating in the network is larger at every point in time. The large separation between the two participation curves demonstrates the success of SOS.

### The relative advantage of SOS depends on the density and message frequency

Figure 2A shows the difference in longevity between mesh and SOS as a function of message frequency and population density. The message frequency ranges from sending 1 to 10 messages per 15 min. The population density is varied by increasing the number of phones in a fixed area. Concretely, the population density is varied from 0.16, representing 100 phones, to 1.28, representing 800 phones.

The primary energy expenditure of mesh networks comes from connecting phones. Therefore, the right side of Fig. 2A—where there are more phones and thus more connections—shows larger advantages for SOS. The primary energy expenditure for SOS comes from relaying, as routes are longer. Therefore, the lower side of Fig. 2A—where fewer messages are sent and relayed—shows larger advantages for SOS. Conversely, the mesh topology performs better in scenarios with low densities and large numbers of messages.

Note that the number of messages for scenarios where the mesh topology outperforms SOS is extreme, with every phone continuously sending multiple messages in each time step. For all other scenarios, SOS outperforms the mesh topology, with the best performance for high phone densities (as in disaster-struck cities) and reasonable volumes of information traffic (which can be technically ensured).

To interpret the densities and relate our simulated world to the real world, we need to make a few assumptions. The transmission range is 5 points, in a 25 × 25 world, while the transmission range in the real world would be around 50 m. Therefore, the simulated world can be assumed to represent approximately an area of 250 m^2^. This means that the simulated densities correspond to a range of densities observed, for example, in cities such as Sydney or Tehran. To have a general idea of where actual cities sit in terms of density, in Fig. 2B, example cities are provided for every density in the legend below. The United Nations Department of Economic and Social Affairs/Population Division, World Urbanization Prospects in 2011 published a report on metropolitan urban cities in the world that are at highest risk of getting affected by natural calamities^53–55^. We show the top 10 cities in the world that have the highest risk of getting affected by five natural calamities: earthquake, tsunami, river flood, storm surge and tropical cyclones.

## Discussion

The increased penetration of mobile phones in remote parts of the world has opened avenues for their use to improve situational awareness during disasters^56,57^. In this contribution, we developed a novel protocol for peer-to-peer communication using a “design for values” approach. The value of participatory fairness is particularly important in emergency situations. Our protocol achieves social fairness by self-organising and adapting its topology to the spatio-temporal context of a disaster situation. In the resulting peer-to-peer network, phones with high battery charge work as hubs, facilitating emergency communication for those citizens who are in immediate danger and have little battery charge to spare. This is in contrast with the generic mesh topology that underlies previously proposed emergency communication solutions. These solutions form so many connections that they do not provide the required functionality over a 72 h period.

It seems that recent developments in emergency communication have focused more on introducing infrastructure to disaster-struck areas than on social innovation and better governance. For example, base stations with Wi-Fi capability may be brought to a disaster area, or unmanned aerial devices can provide connectivity^22,58–61^. As mentioned above, however, the logistics of disaster response typical implies delays for such solutions, while delays are often deadly. That is why a solution such as SOS is needed, which works over an extended period of time even in the absence of recharging opportunities. Still, we think that every kind of emergency communication solution has its role to play. Generic mesh itself may transmit messages faster than SOS, because there are fewer hops in between. In situations where batteries can be recharged, generic mesh may therefore be preferable to SOS. Hence, it might be helpful if the communication protocol itself would adjust to the situation at hand. Dynamic decentralized switching between communication protocols is something we are currently looking into.

However, the importance of the SOS approach is steadily increasing, as it works best in densely populated, urban areas. Currently, over half of the world’s population is living in cities, and this proportion is still growing. Also, the population density of cities is increasing, and so is the disparity of resources^62^.

In the simulations that we used to illustrate differences between the SOS and mesh communication networks, battery charge was randomly distributed. For mesh, people with high battery charge phones will be able to send messages for longer than those with low battery charge phones. Low battery charge phones will quickly lose the ability to communicate, typically long before the crucial 72 h period is over. With SOS, however, those people with high battery charge phones will increase the communication opportunities of those with low battery charge phones, thereby strengthening the social fabric and resilience^63^.

Routing for energy efficiency is not new, as there have been many proposals to prolong battery life, also in ad hoc networks. However, we are not primarily interested in energy efficiency here, but rather in a socially fair distribution of communication opportunities, which benefits individuals and social communities alike. If the energy required to maintain an inclusive communication network is shared, this produces a public good, where everyone can benefit from the increase in collective action and collective intelligence this enables.

Our definition of participatory fairness differs from game theoretical fairness definitions, and is more closely aligned with the literature on equity and justice. Rather than focusing on a competitive exchange, where parties can justly or unjustly gain advantages at the cost of others, we interpret fairness in the context of a redistribution of opportunities in times of need.

The requirement of participatory fairness has important implications for the design of peer-to-peer networks, and improves emergency communication in two ways: (i) it enhances communication opportunities for a large number of people and (ii) it considerably extends the time period over which peer-to-peer communication can be maintained.

Finally, citizens are not interchangeable commodities, of course: Not all citizens will contribute in the same way. Different people have different requirements, but can also offer different skills and contributions^64^. Thus, it is important that everyone stays connected. Furthermore, abilities and requirements are not static, as they may change over time. By adapting to changing circumstances, and maximizing the strengths of each, one can empower individual citizens and community resilience, without forgetting those that are in need of support. Our communication protocol does just that.

## Methods

The performance of SOS was compared to a mesh communication topology through modeling and simulation in terms of longevity, traffic adaptivity, battery charge inequality, and phone participation (see the SI for details of the experimental setup).

### Populating the model and simulating behavior

To examine the performance of both mesh and SOS communication networks, and to make comparative analyses, a mesh-based topology and a SOS-based topology were simulated in two separate agent-based models. The models assume a two-dimensional torus-shaped world and populates it randomly with nodes. Each node denotes a mobile phone that moves independently and in a random walk. Two nodes can communicate when there is a link between them. It is created when they are within transmission range of each other. If there is no direct link, nodes can communicate through intermediate nodes. Each node has a limited transmission range. When the nodes move out of transmission range they lose connection, i.e., the corresponding link breaks.

There is no limitation on the number of connections a node can form. Nodes have fixed battery charge that depletes once they start making connections and sending messages. Participating nodes are not assigned any roles as they join the network. As the network formation begins and the number of participating nodes increases, different roles are assigned to nodes automatically and dynamically to maintain connectivity and energy efficiency.

Nodes communicate directly or through multiple nodes/hops relaying messages. Algorithm 3 (see SI) is used for routing. In each model, all nodes send a message to a randomly selected node present in the model for receiving the message. The number of messages sent by each node is a model parameter and can vary from 1 to 10. In case no route is found, each node saves the message as “pending”.

### Model limitations and future work

SOS could be implemented either as part of an operating system or simply as an application running on a smartphone, thereby putting an additional governance layer on top of the technical ad hoc network functionality. Hence, the overlay network can be established and operated independently of service providers. The model also considers the loss of battery charge associated with other processes running on phones. Such processes would be restricted based on bandwidth needed. For example, text messages and similar low bandwidth communication would be prioritised, while pictures and videos would be restricted or sent with lower quality to spare bandwidth.

Communication devices have individual characteristics that might add further parameters for investigation. For example, under real circumstances, the diversity of phones implies different capabilities in-built in each of them, such as the amount of memory available for higher performance and traffic management. Our present model has not incorporated such traffic management and storage capacities. Including them might allow for additional refinements to the algorithms. However, these parameters are currently out of scope for this article and are an interesting subject for future research.

## Supplementary Information

Supplementary Information.

Supplementary Movie S1.

Supplementary Movie S2.

Supplementary Movie S3.

## Data Availability

All data and models necessary to interpret, replicate and build upon the methods or findings reported in the article are available upon request.
