# Supplementary material for: Introducing participatory fairness in emergency communication can support self-organization for survival
Source: Sci Rep. 2021 Mar 30;11:7209. doi: 10.1038/s41598-021-86635-y (PMC8010119; doi:10.1038/s41598-021-86635-y)
Supplement: Supplementary file 1 — Supplementary Information. [file 41598_2021_86635_MOESM1_ESM.pdf]

Supplementary Material: Introducing participatory fairness in emergency communication can support self-organization for survival

Indushree Banerjee[1], Martijn Warnier[1], Frances M T Brazier[1] & Dirk Helbing[2]

[1]TU Delft, Systems engineering and Simulations, Delft, 2628 BX, The Netherlands
[2]ETHZ, Computational Social Sciences, Zürich, 8092, Switzerland

March 16, 2021

Introduction

In systems engineering, the first and foremost step is to specify requirements that help to define the purpose and attributes of the system being designed. These define the tasks of the system, qualities or attributes it must have and specific conditions under which it should perform those tasks[1, 2].

Functional and non-functional requirements

Current ad hoc communication systems (such as TeamPhone, RescueMe, HelpMe, ServalMesh, Firechat, LifeNet, SENSE-ME, StemNet [3, 4, 5, 6, 7, 8, 9, 10]) meet the functional requirements of an infrastructure-less ad hoc communication system that allows people to connect. These include:

1. **Connectivity:** All citizens should be able to connect using their phones to their neighboring phones to form an ad hoc network.
2. **Reliability:** All messages that are sent must be delivered to the intended receiver.

There are also certain non-functional requirements that need to be considered. These are functionalities that must be performed under certain constraints, such as:

1. **Scalability:** The performance of the network must not be affected by density changes in the population.

2. **Durability:** All message exchange should be reliable for at least 72 hours (after the sudden onset of a disaster) for every individual.

3. **Participatory fairness:** All phones despite disparity of battery charge should be able to communicate.

Our goal was to meet the above non-functional requirements along with the functional requirements. In addition to the results in main manuscript, the proposed approach is also reliable and scalable [11]. In the design process, it was decided to deliver the functional requirements as the emergent property of the system. Emergent properties of a system are the properties that are not exhibited by the components of a system but result from the particular kinds of interactions. Thus, a connected network that provides a durable and fair message exchange in our design is an emergent property. This led to the following design choices explained in the next section.

Design choices

In order to achieve the non-functional requirements, it was important to make certain design choices. These choices were in regard to certain trade-offs. These are explained below:

1. **Spatial awareness and context adaptivity**
   The dynamic nature of a disaster area represents various challenges. For example, the number of people with phones and the battery charge in these phones changes all the time. To address this, it was decided that the protocol will be a set of algorithms that focus on local interaction based on context information.

2. **Autonomous self-healing**
   The mobility of people and phones leaving the network can lead to dropped messages as routes can be lost. Therefore, self-healing properties need to be considered. Thus, autonomous event-driven reconfiguration had to be designed.

3. **Minimizing connections while maintaining functional and non-functional requirements**
   In a traditional infrastructure-less ad hoc network, energy is lost in connecting, sending, receiving and relaying through phones. Each of these operations needs to be optimized. Formation of a network using context-adaptive self-organisation is a better solution to reduce the connections cost, specially in densely populated areas. This trade-off between connection cost and relaying cost has been discussed in detail in main manuscript.

Two distributed algorithms were designed with the criteria that each phone (Phone and node are used interchangeably) with limited information will have only local interactions with other phones to connect and self-configure in order

to fulfill the functional and non-functional requirements. Each phone engages in limited information exchange with other phones in its transmission range to be spatially aware (regarding potential phones to connect with) and self-aware (regarding its own battery charge).

Algorithms and pseudocode

There are two algorithms that are part of this protocol. Algorithm 1 defines the connection procedure by forming connections based on contextual information information. Algorithm 2 determines the event-driven reconfiguration to maintain autonomous self-organisation. As each phone follows these two algorithms, they follow a cycle of monitoring spatial changes, analyzing context, planning next steps, and executing decisions based on this information. Monitoring, analyzing, planning and executing are the four fundamental steps of MAPE cycle [12]. Following a MAPE cycle is critical for designing autonomous systems and in this case leads to the emergence of a context-adaptive self-organised network. Algorithm 1 and algorithm 2 are used to create, and adapt the topology of the network as the context (spatial-temporal and resource) changes in their environment. To model and simulate the communication part of the network we design algorithm 3. Algorithm 3 is for message routing and the pseudocode is presented in the modeling section separately as it is not part of the main protocol and utilizes a gradient-based routing protocol [13]. Algorithm 1 and 2 are explained in detail in the following section. The illustration of algorithm 1,2 and 3 for simulation and modeling is presented in Fig. 1.

Connection mechanism

The process starts with each phone on its own, which is not connected to any other phone in its transmission range. As each phone turns on their Bluetooth, they start discovering phones in their transmission range r. Each phone then collects information of its surrounding by creating an information tuple t. The spatial information stored in t is presented in Table 1, and it consists of their own unique identifier (U_{id}), subtree identifier (S_{id}) and battery charge left (e). The phone also maintains a list of its connections (l_v). Initially l_v is empty as a phone is not connected to anyone. As the phones start making connections this list grows.

Table 1: The information stored in t

Information	Type
U_{id}	Unique identifier of the phone
S_id	Subtree identifier = connected network number
e	Battery charge left

Initially the subtree identifier is set to the unique identifier of the phone to represent that the phone is disconnected from any network. As a phone

informs its presence and starts to explore possible connections, it also starts receiving the t from other phones in its transmission range. Based on this spatial information, the phone then decides a favourable or preferred choice of connection. This connection is based on the battery charge of other phones. The phone considers the energy of all the potential phones in the range and sorts them in decreasing order of battery charge. The potential phone with the highest energy is selected and the connection-initiating phone compares their subtree identifier. If they do not match, it confirms that both these phones belong to different networks and then these phones connect and change their subtree identifier to match. Once connection is established, both phone populate their l_v with the connection they made. This procedure ensures that there are no redundant routes between nodes. Different subtree identifiers indicate that phones belong to different networks. If the subtree identifiers match it indicates that the nodes are connected through different phones in the same network. By matching subtree identifiers, redundant routes between nodes are prevented.

Algorithm 1 Connection Procedure followed by each node in Transmission range r

$t \leftarrow U_{id}, e, S_{id}, l_v$
$U_{id} = S_{id}$ ▷ Node A sets its unique identifier to its subtree identifier signifying it is not connected to any network
▷ Node A turns on its Bluetooth and scans for neighbors within r
▷ list O_l is a temporary list generated to store information of other phones within r
if O_l != *null* **then** ▷ Other phones found within r
    $O_l \leftarrow t_D, t_C, t_B, ...$ ▷ Put the information tuple of neighboring phones
    $O_l \leftarrow t_{B_e} > t_{D_e} > t_{C_e}, ...$ ▷ Sort O_l in decreasing battery charge
EndIf
while O_l != *null* **do** ▷ Select neighbor to connect from the list
    if $S_{id}(A)$!= $S_{id}(B)$ **then** ▷ Compare the S_{id} of Node A with the S_{id} of Node B
        $S_{id}(A) = S_{id}(B)$ ▷ Connect and equalize subtree identifier
        $l_v \leftarrow B$ ▷ Include the new connection in list l_v, initially empty.
        Break
    EndIf
EndWhile
$O_l \leftarrow null$ ▷ Empty O_l

Let's assume citizen A is disconnected from communication infrastructures and would like to form a network or get connected to an ad hoc network. A switches on its Bluetooth and starts to explore other phones around in its transmission range. The phone of A finds other citizens (B,D) also trying to form a network or get connected. These phones exchange their information tuple that consists of their own U_{id}, their S_{id} (which at this point will be set to their unique identifier, since they are all disconnected), and their own battery charge

(e). They also have a empty list of connected neighbors (l_v), which they will populate once they start forming connections.

Imagine the battery charge in phones of the citizens is in order A >D >B. Thus A has the highest energy of all of them. The phones B and D select A to connect since it can afford multiple connections. Now phones B and D change their subtree identifier to the subtree identifier of A, representing that they are now part of the same network. Between B and D there is no direct connection since they are connected via A. They know this as they both have the same subtree identifier. This prevents the formation of redundant routes reducing connection cost and ensuring low node degree for less battery charge phones. Since there is local information exchange in a distributed manner, a context-adaptive self-organised network emerges.

These connection patterns and the topology keep changing as phones move in and out of the transmission range. Suppose citizen M comes in the range of this network and has higher battery charge than A. Then M becomes central as other phones switch to phone M. This way the traffic is diverted through a separate set of phones and prevents the exploitation of certain specific phones. Local information exchange ensures that the energy lost in this exchange is limited and maintains the trade-off between adaptivity and energy consumption.

This connection procedure results in a fully connected network with a dynamic topology that ensures participatory fairness and energy efficiency. A network structure emerges with central phones being high in energy and pushing low energy nodes to the edge of the network. To improve the performance, phones remove connected phones with exhausted battery and connect to new phones whenever the network is unable to send or receive information. Thus this self-organizing adaptive protocol allows mobile phones to interact with their neighbors in a distributed mechanism to collaboratively achieve connectivity, robustness, scalability and reliability as emergent properties of the entire system.

Event-driven reconfiguration: Adaptive reconfiguration and relabeling

The purpose of the protocol is to maintain reliability and robustness despite link failures and phones leaving due to loss of battery charge. Additionally the topology must adapt to ensure that the loss of energy for relaying messages is distributed among various high energy nodes. This is achieved by event-driven reconfiguration. Let's assume citizen A has a set of pending messages in his phone that it needs to forward/relay to the next hop. The algorithm then triggers an event-driven reconfiguration. This results in A following algorithm 2.

The phone first looks into its list of connected phones, l_v. Let's say A is connected to citizen B, C, D, E, M and N. Since citizens are walking around and are not static it is quite possible that some of them are out of range. Additionally, they are losing battery charge, meaning that some of them may now be below the acceptable level for getting connected. Therefore A updates its l_v by removing links with citizens that are out of its transmission range (D and

M) and removing links with citizens with phones that have almost negligible battery left (C and E). If all connections were removed during the previous steps then the phone of A also changes its own subtree identifier back to its own unique identifier and it recursively asks all still connected phones to change their subtree identifier back to the caller's unique identifier i.e., to the unique identifier of node A. This ensures that all subnetworks that emerge from this step have unique identifiers, thereby maintaining consistency.

However, to utilize mobility and maintain connections, node A looks for possible new neighbors or phones with high energy. If new citizens with higher-battery-charge phones come into transmission range, A follows the same connection procedure described before in Algorithm 1 to prevent redundant routes while still getting connected and forming new connections or become part of a network.

Algorithm 2 Event-driven reconfiguration followed by a node A

$l_v \leftarrow t_B, t_C, t_D, t_E, t_M, t_N$
  ▷ list l_v is newly generated by exploring other nodes within transmission range
Remove links with nodes with low battery charge
$l_v \leftarrow t_B, t_D, t_M, t_N$
  ▷ list l_v is updated by removing nodes C and E
Remove links out of transmission range r
$l_v \leftarrow t_B, t_N$
  ▷ list l_v is updated by removing nodes D and M
Relabel node subtree identifier using:
procedure RELABEL(Node)
  if S_{id} != $U_{id}(A)$ **then**
    Set $S_{id} = U_{id}(A)$
    RELABEL(connected nodes)
  else break
Connect to new nodes by following **Algorithm 1**

This leads to the emergence of a new topology every time there is an event-driven reconfiguration, which is not only optimized for finding new routes for sending and receiving messages but also more energy efficient as different high energy phones act as hubs. As every phone follows these three algorithms autonomously with distributed information exchange, the network remains robust, scalable and reliable for changing density and energy availability.

Method: Modeling and simulation of mesh network and SOS

As described in the main manuscript, both mesh and SOS were modelled in NetLogo. Below, more detail is provided on the implementation.

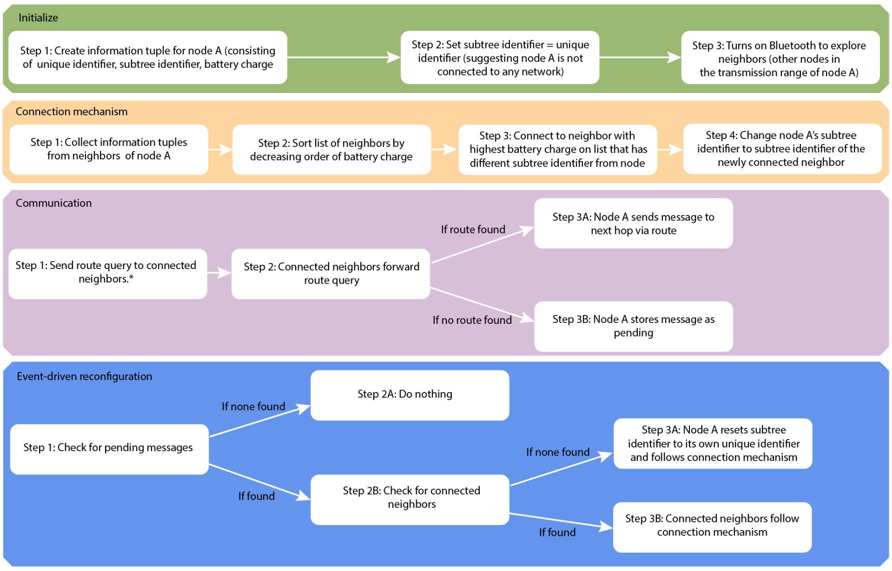

Figure 1: The figure represents the detailed working of the algorithm 1 (green and orange), algorithm 2 (purple) and algorithm 3 (blue). Note that this is done by an independent routing protocol (outside scope of the paper)

Populating the model and simulating behavior

To examine the performance and do comparative analysis of both mesh and SOS, both topologies were simulated in two separate agent-based models. The model takes a two-dimensional torus-shaped world and populates it randomly with nodes. Each node denotes a mobile phone that moves independently and in a random walk. Two nodes can communicate when there is a link between them, which gets created when they are in the transmission range of each other. If there is no direct link, nodes can communicate through intermediate nodes. Each node has a transmission range. When the nodes moves out of transmission range they lose the connection link. This helps in investigating the effect of mobility on performance, scalability and robustness of the network.

There is no limitation on the number of connections a node can form. Nodes have fixed battery charge that depletes once they start making connections and sending messages. As the simulation starts, every node follows algorithm 1 that lead to the emergence of an ad hoc network. Participating nodes are not assigned any roles as they join the network. As the network formation begins and the number of nodes participating increases, different roles are assigned to them automatically and dynamically to maintain connectivity and energy efficiency.

Nodes communicate directly or through multiple nodes/hops relaying messages. To maintain participatory fairness, the traffic keep flowing through dif-

ferent nodes.

Routing of messages

In each model, all nodes send a message to a randomly selected node present in the model for receiving the message. The number of messages sent by each node is a model parameter and can vary from 1 to 10. Assume citizen A wants to send a message to citizen Z. If citizen A is not connected to Z directly, A sends a route enquiry to all its connected phones using algorithm 3.

If there are no direct connections, relaying phones then forward this enquiry to their own connections. This route enquiry can have two results. First if there is a route, A is notified and A forwards it to the next hop or relay. If at some point during relaying one of the relaying phones does not find a route, it stores the message as a pending message. Once it gets a route, it forwards the message to the receiver Z. This is true for mesh topology and is thus implemented in the simulation as such.

Algorithm 3 Communication: routing of messages

```
Sender sends a route inquiry
if No Route found then
    if Connected to a Network then
        Ask connected neighbors to Reconfigure
    else Node is Not connected to any Network
        Self Reconfigure
else Route found
    Send information to next node in the route
EndIf
```

However, for SOS the model follows algorithm 3. When there are no routes A saves the message as pending. Next A checks if it is disconnected or if it has connections. In case it is disconnected, A reconfigures following algorithm 2. If A has connections, A triggers algorithm 2 for its connections. The communication algorithm pseudocode is presented in algorithm 3. A gradient-based routing protocol [13] is assumed for routing.

Modeling changes in battery charge

To ensure that energy efficiency is achieved, it is important to consider each factor associated with losing battery charge while participating in a network. The first consideration was to choose between available interfaces for communication i.e. either Bluetooth low energy (BLE) or Wi-Fi. Research suggests that BLE was 30% more energy efficient than Wi-Fi. Moreover, BLE is a standard interface that is available in every phone making our approach more easy to deploy in countries where the population cannot afford smartphones.

The battery costs associated with sending, receiving, relaying and forming connections were taken from the literature [14], and are presented in Table 2. A

variety of phones is modeled: We simulated a combination of high-end phones such as Apple iPhones (with full battery charge capacity of 3000mAh) and low-end budget phones, non-smartphones (with full battery charge capacity of 2000mAh).

**Activity**	**Power draw (mA)**	**Duration (mS)**	**Bytes**	**Time*Current (mAmS)**
Wakeup Preprocessing	5	1000		5000
Receiving (Rx)	22	1120	140	24620
Inter Frame Space (IFS)	15	150		2250
Transmission (Tx)	28	1120	140	31360
Post Processing	8	1400		11200
Total Time		4790		
Total Time * current				74450

Table 2: Energy consumption associated with sending and receiving a single SMS over a BLE connection [14, 15]

For both the models, phones can form a direct connection with other phones if they are in transmission range. In mesh all phones connect that are in range, without consideration of the battery charge of phones. For mesh, if phones move out of range, they remove links and if phones no longer have energy they leave the network.

For simulating SOS, algorithm 1 and algorithm 2 are followed. The sending and relaying is same in both the topologies and hence kept the same across models. The real difference lies in the event-driven reconfiguration that is only implemented in SOS by following algorithm 3.

Apart from this, both simulations perform the same tasks and are initiated with an equal number of nodes and equal battery charges of the phones for the sake of comparison. For SOS, the cost associated with event-driven reconfiguration is taken into consideration and calculated as per Table 2.

Evaluation

Impact of transmission range variability

Additionally, we have performed experiments with variable transmission ranges (specifically, a uniform distribution ranging from 5 to 8 units). In its present form, the algorithm also works given such heterogeneity, as the decision whether to connect or not, is on the basis of battery availability around a node. The transmission range impacts the number of possible connections a node can choose from. Nodes with higher transmission range see a larger selection pool increasing their chances to getting connected to higher battery charge phones. This increases the overall network lifetime, as shown in the Fig. 2. For nodes with smaller transmission range the pool of possible connections to choose is smaller; However, the rules remain the same, i.e., connect to the highest battery phone in the vicinity. Given the context-adaptive nature of our approach, which automatically self-heals and self-organizes the network, fairness is not impacted as an emergent global property (see Fig 3.).

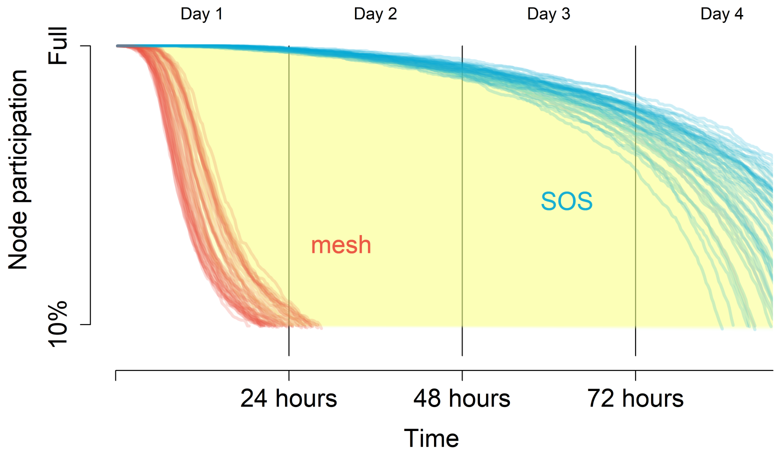

Figure 2: Node participation over 72 hours for the mesh network (red) and for SOS (blue) for varying transmission range

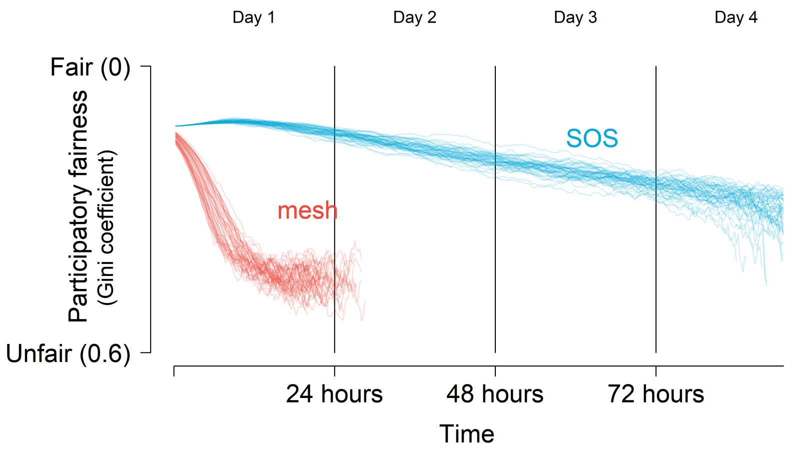

Figure 3: Development of battery charge inequality(Gini coefficient [51]) over 72 hours for the mesh network (red) and for SOS (blue) for varying transmission range

Phase diagram

In the phase diagram, Fig. 2 of the main text, the ratio of the longevity is plotted for a single run of both SOS and mesh, for 8x10 combinations of node density and message frequency.

To get an estimate of the sampling variability, five combinations of node density and message frequency were repeated 100 times. Density plots for these 100 runs are displayed in Supplementary Fig. S2. In each density plot, a vertical line is provided to show the SOS and mesh longevities that were used to create the phase diagram. Although the inter-run variability is different for different combinations of factors, the inter-run variability is small compared to the difference between the two protocols.

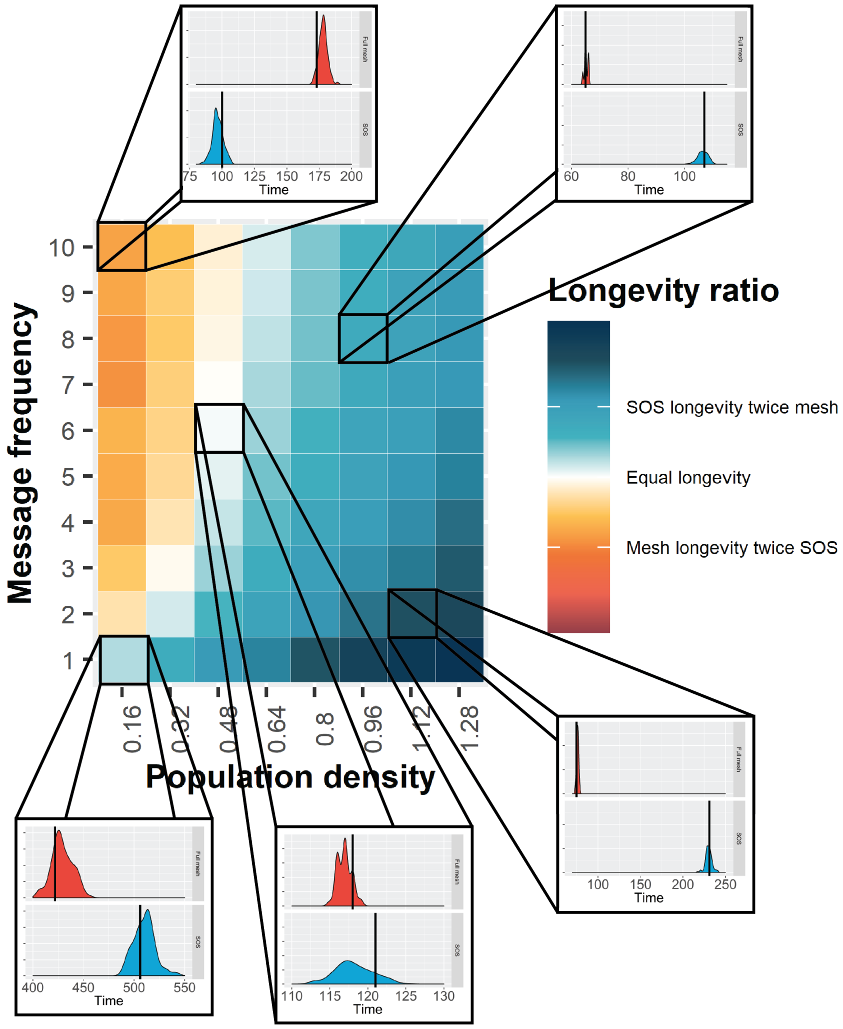

Figure 4: Phase diagram

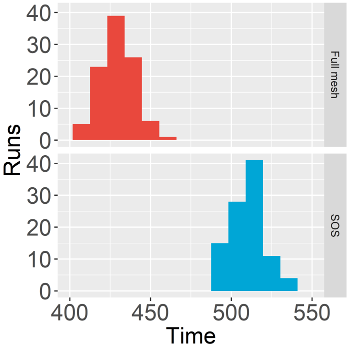

(a) 100 nodes sending 1 message

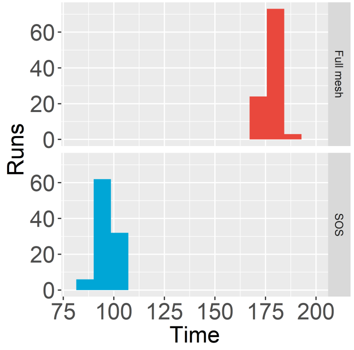

(b) 100 nodes sending 10 messages

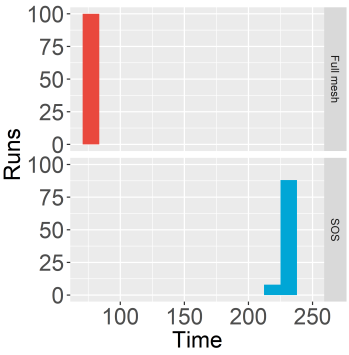

(c) 700 nodes sending 2 messages

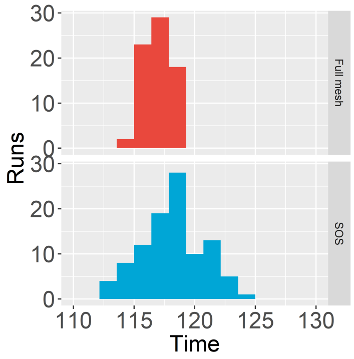

(d) 300 nodes sending 6 messages

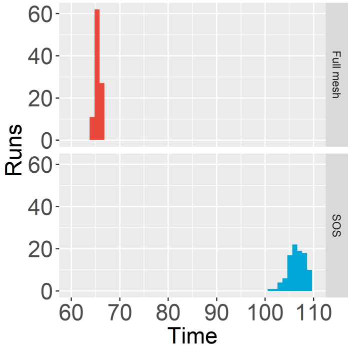

(e) 600 nodes sending 8 messages

Figure 5: Histogram of longevity across a 100 runs for mesh (red) and SOS (blue), with varying density and message frequency

Movies

Illustration of both SOS and mesh topology evolving network.

It is a simulation of 500 phones sending and receiving messages. We demonstrate the energy loss and node participation.

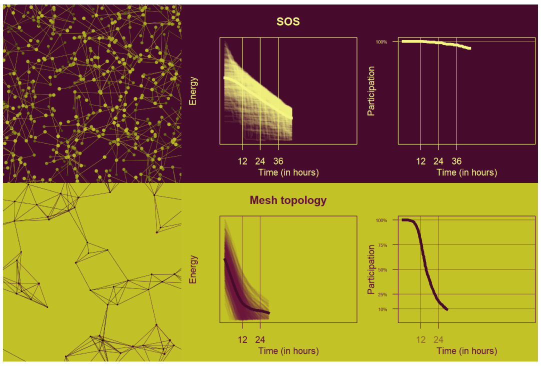

Figure 6: Screenshot of movie S1

SOS: Illustration of the SOS evolving network.

It is a simulation of 500 phones sending and receiving messages. We demonstrate the energy loss and betweenness centrality of three phones. These are phones with high (green), medium (yellow) and low (red) initial battery charge.

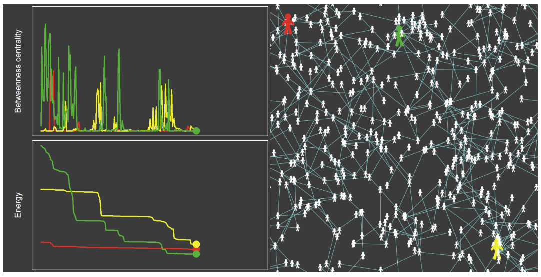

Figure 7: Screenshot of movie S2

Mesh: Illustration of a mesh topology evolving network.

It is a simulation of 500 phones sending and receiving messages. We demonstrate the energy loss and betweenness centrality of three phones. These are phones with high (green), medium (yellow) and low (red) initial battery charge.

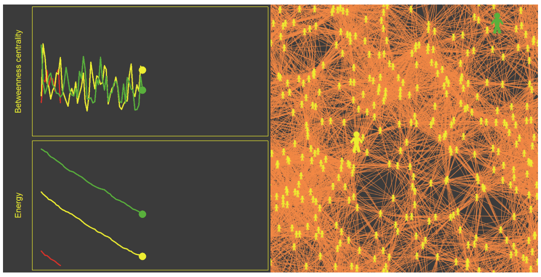

Figure 8: Screenshot of movie S3

References

[1] Somerville, I. *Software Engineering* (Pearson, 2016), 10th edn.

[2] Ryan, M. & Faulconbridge, R. *Systems engineering practice* (Argos Press, 2014).

[3] Lu, Z., Cao, G. & La Porta, T. Teamphone: Networking smartphones for disaster recovery. *IEEE Transactions on Mobile Computing* **16**, 3554–3567 (2017).

[4] Sun, J., Zhu, X., Zhang, C. & Fang, Y. Rescueme: Location-based secure and dependable vanets for disaster rescue. *IEEE Journal on Selected Areas in Communications* **29**, 659–669 (2011).

[5] Miranda, K., Molinaro, A. & Razafindralambo, T. A survey on rapidly deployable solutions for post-disaster networks. *IEEE Communications magazine* **54**, 117–123 (2016).

[6] Aloi, G., Briante, O., Di Felice, M., Ruggeri, G. & Savazzi, S. The sense-me platform: Infrastructure-less smartphone connectivity and decentralized sensing for emergency management. *Pervasive and Mobile Computing* **42**, 187–208 (2017).

[7] Legendre, F., Hossmann, T., Sutton, F. & Plattner, B. 30 years of wireless ad hoc networking research: what about humanitarian and disaster relief solutions? what are we still missing? In *Proceedings of the 1st International Conference on Wireless Technologies for Humanitarian Relief (ACWR'11)*, 217–217 (2011).

[8] Aloi, G. *et al.* Stem-net: How to deploy a self-organizing network of mobile end-user devices for emergency communication. *Computer Communications* **60**, 12–27 (2015).

[9] Mehendale, H., Paranjpe, A. & Vempala, S. Lifenet: A flexible ad hoc networking solution for transient environments. In *Proceedings of the ACM SIGCOMM 2011 Conference*, SIGCOMM '11, 446–447 (2011).

[10] Gardner-Stephen, P. *et al.* The serval mesh: A platform for resilient communications in disaster & crisis. In *2013 IEEE Global Humanitarian Technology Conference (GHTC)*, 162–166 (IEEE, 2013).

[11] Banerjee, I., Warnier, M. & Brazier, F. M. Self-organizing topology for energy-efficient ad-hoc communication networks of mobile devices. *Complex Adaptive Systems Modeling* **8**, 1–21 (2020).

[12] Kephart, J. O. & Chess, D. M. The vision of autonomic computing. *Computer* **36** (2003).

[13] Yi, S., Heo, J., Cho, Y. & Hong, J. Peach: Power-efficient and adaptive clustering hierarchy protocol for wireless sensor networks. *Computer communications* **30**, 2842–2852 (2007).

[14] Lindh, J., Lee, C. & Hermes, M. Measuring bluetooth low energy power consumption. Tech. Rep., Texas Instruments (2017).

[15] Kamath, S. & Lindh, J. Measuring bluetooth® low energy power consumption. Tech. Rep. Application Note-AN092, Texas Instruments (2010).
